# Aging But Not Age-Related Hearing Loss Dominates the Decrease of Parvalbumin Immunoreactivity in the Primary Auditory Cortex of Mice

**DOI:** 10.1523/ENEURO.0511-19.2020

**Published:** 2020-05-08

**Authors:** Meike M. Rogalla, K. Jannis Hildebrandt

**Affiliations:** Department of Neuroscience, Division of Auditory Neuroscience, and Cluster of Excellence, Hearing4all, Carl von Ossietzky University, Oldenburg 26129, Germany

**Keywords:** age-related hearing loss, aging, mouse primary auditoy cortex, parvalbumin

## Abstract

Alterations in inhibitory circuits of the primary auditory cortex (pAC) have been shown to be an aspect of aging and age-related hearing loss (AHL). Several studies reported a decline in parvalbumin (PV) immunoreactivity in aged rodent pAC of animals displaying AHL and conclude a relationship between reduced sensitivity and declined PV immunoreactivity. However, it remains elusive whether AHL or a general molecular aging is causative for decreased PV immunoreactivity. In this study, we aimed to disentangle the effects of AHL and general aging on PV immunoreactivity patterns in inhibitory interneurons of mouse pAC. We compared young and old animals of a mouse line with AHL (C57BL/6) and a mutant (C57B6.CAST-*Cdh23^Ahl+^*) that is not vulnerable to AHL according to their hearing status by measuring auditory brainstem responses (ABRs) and by an immunohistochemical evaluation of the PV immunoreactivity patterns in two dimensions (rostro-caudal and layer) in the pAC. Although AHL could be confirmed by ABR measurements for the C57BL/6 mice, both aged strains showed a similar reduction of PV^+^ positive interneurons in both, number and density. The pattern of reduction across the rostro-caudal axis and across cortical layers was similar for both aged lines. Our results demonstrate that a reduced PV immunoreactivity is a sign of general, molecular aging and not related to AHL.

## Significance Statement

Deficiency of sensory functions is one of the major detriments of aging. In hearing, aging affects both the periphery and inhibitory circuits in the central system, resulting in hearing loss and altered perception. Centrally, the major subclass of inhibitory interneurons [parvalbumin (PV)^+^] shows reduced PV immunoreactivity, which is believed to be related to altered inhibition. Identifying the factor that dominates this decline is important to understand molecular aging in the central auditory system. Here, we demonstrate that the decreased PV immunoreactivity in the primary auditory cortex (pAC) of mice is dominated by general aging rather than age-related hearing loss (AHL), suggesting that altered cortical inhibition in the auditory system may not be secondary to peripheral changes, but a consequence of aging per se

## Introduction

Age-related hearing loss (AHL), also referred to as presbycusis, is one of the most common sensory impairments worldwide, approximately affecting one third of adults above 65 years in forms of progressive loss of auditory function (Roth et al., 2011; Loughrey et al., 2018).

On the physiological level, the loss of hearing during aging is usually related to a loss of sensory hair cells or a decline of spiral ganglion cells (Frisina et al., 2016). Such age-related morphologic changes have not only been observed in humans, but also in laboratory rodents, especially in mice (Hequembourg and Liberman, 2001; McFadden et al., 2001; Li et al., 2002) which therefore have been suggested to be a suitable model of AHL (Gratton and Vázquez, 2003; Bowl and Dawson, 2015).

The decline of peripheral signal transmission has been linked to subsequent pathologic changes within the central nervous system, especially in the primary auditory cortex (pAC; Eckert et al., 2012). Alterations in inhibitory circuits of the pAC have been linked to hearing loss and the aged auditory system (Kotak et al., 2005; Takesian et al., 2012; Peelle and Wingfield, 2016). The largest class of cortical GABAergic interneurons, the parvalbumin (PV) neurons, are believed to play an important role when it comes to loss-of-input dependent changes in the neuronal inhibitory circuits of pAC. Several studies reported a relationship between AHL and a decline in PV immunoreactivity in the rodent auditory cortex (de Villers-Sidani et al., 2010; Martin del Campo et al., 2012; Brewton et al., 2016), whereas others report a decline in PV immunoreactivity for several cortical areas, unrelated to any decline in sensory function (Miettinen et al., 1993; Ueno et al., 2018). Because of these contrary findings, it remains elusive whether a decline of PV immunoreactivity in pAC is a result of progressive AHL and the loss of input or if it is just a general phenomenon in the aging central (auditory) system.

In this study, we aimed to resolve the effect of aging and AHL on the PV immunoreactivity in the pAC of mice. To this end, we used a mouse line with a rapid, progressive development of AHL (C57BL/6) and the mutant C57B6.CAST-*Cdh23^Ahl+^*, a congenic strain that carries the wild-type allele of *Cdh23,* producing C57BL/6 mice that are not vulnerable to AHL (Johnson et al., 1997; Keithley et al., 2004; Mock et al., 2016). We compared young and old animals of both lines according to their hearing status by using auditory brainstem response (ABR) measurements and an immunohistochemical evaluation of the PV immunoreactivity patterns in two dimensions (rostro-caudal and layer) of the pAC.

## Materials and Methods

### Experimental groups

All animal experiments were performed in accordance with the animal welfare regulations of Lower Saxony and with the approval from the local authorities (State Office for Consumer Protection and Food Safety/LAVES, permission number 33.9-43502-04-13/1271).

In total, 43 mice were used in this study, of which 21 were used for ABR measurements only, 15 in histology only, and seven animals in both.

Male C57BL/6J (stock number 017320; RRID: JAX:017320) mice were used to serve as an animal model with AHL. In contrast, male C57B6.CAST-*Cdh23^Ahl+^* (stock number 002756; RRID: JAX:002756) mice were used as an animal model without the development of AHL. Both strains were in-bred animals, originating from purchased breeding pairs (The Jackson Laboratory) and kept in small colonies with *ad libitum* access to water and food in the local animal facility under standardized conditions until terminal experiments.

The age groups were designed as follows: young animals (10–12 weeks) of both strains (young*^B6^*, *n* = 11) and young*^B6.CAST^*, *n* = 7) and aged animals (12–15 month) with AHL (aged*^B6^*, *n* = 13) and without (aged*^B6.CAST^*, *n* = 12; for details, see Table 1).

**Table 1:** Assignment of animals to the groups according to hearing status and age

Group	Age	Histology	ABR	Double	Total
Young^B6^	10–12 weeks	6	6	1	11
Young^B6.CAST^	10–12 weeks	2	9	1	7
Aged^B6^	12–15 months	8	7	2	13
Aged^B6.CAST^	12–15 months	6	9	3	12
Sum		22	28	7	43

### Evaluation of hearing status, ABR

Animals were anesthetized with ketamine (initial 10 mg/kg, maintenance 2.5 mg/kg) and medetomidine (initial 0.083 mg/kg, maintenance 0.01 mg/kg). The state of anesthesia was checked in regular intervals, if needed anesthesia was toped up with the maintenance dose (typically every 1.5 h). Needle electrodes were placed subcutaneous with the recording electrode at the neck and the reference electrode at the vertex. The electrode signal was bandpass filtered (300 Hz to 30 kHz) and amplified by an ISO-80 Bio-amplifier (World Precision Instruments), before A/D-converted through a Fireface UC 24-bit sound device at a sample rate of 96 kHz. The same device was used for stimulus delivery. Stimuli were created by a custom MATLAB application. The sound was binaurally delivered directly into the ear canal of the animal through horns that were attached to Vifa/Peerless XT-300 K4 loudspeakers. The system was calibrated before recording using small microphones (Knowles FG-23329) that were inserted into replicas of mice ear canals.

In order to determine hearing thresholds at different tone frequencies, tone pips (10 ms, 2-ms cosine ramps at onset and offset) were presented binaurally at 4, 8, 12, 16, 20, 24, and 30 kHz. The intensity was varied between 35- and 95-dB SPL in steps of 5 dB. Interstimulus intervals were randomly chosen from the interval 50–150 ms. The sequence of stimuli was random. An automatic online artifact rejection algorithm discarded trials with muscle potential artifacts according to a preset threshold. ABR data were recorded continuously and saved for offline evaluation.

### Histology

Animals were injected with a lethal overdose of pentobarbital (Narcoren, Boehringer Ingelheim) and transcardially perfused with phosphate buffer (PB; pH 7.4) followed by fixative (4% paraformaldehyde in PB). The brains were removed and kept in immersion fixative overnight at 4°C, followed by four rinsing steps the next day (PB). For cryoprotection, brains were stored in 30% sucrose solution in PB for 2–3 d at 4°C. After complete saturation, brains were frozen using tissue freezing medium (TFM-5, TBS) and stored at −20°C.

Coronal frozen slices (25 µm) containing pAC were cut (CM 1950, Leica) in series of 4. A mark was placed subcortically into the right hemisphere using a cannula for poststaining distinctness. Slices of the first series were directly mounted on gelatin-coated object slides and air-dried for Nissl staining. Remaining slices of series 2–4 were stored in a 24-well plate/series, containing cryoprotection solution (30% ethylenglycol and 30% sucrose in PBS) and stored at −20°C until immunohistochemistry.

### Nissl staining

The total number of cortical neurons may decrease during aging and this effect may be stronger in animals suffering from hearing loss. To account for this, an evaluation of the total number of neurons in the pAC, a standard Nissl protocol using 0.1% cresyl violet in watery solution was applied. After staining, object slides were rinsed in distilled water, followed by differentiation and complete dehydration with ascending alcohol concentrations. After clearing with xylol, object slides were coverslipped using rapid non-aqueous mounting medium (Entellan, 107960, Merck).

### PV immunohistochemistry

The slices for visualization of PV^+^ interneurons were treated free floating. Slices of one series were rinsed 5 × 5 min in PBS (pH 7.4), followed by 10-min permeabilization in 0.5% Triton X-100 in PBS at room temperature. To prevent nonspecific binding, epitopes on the tissue were blocked with 10% normal goat serum in PBS for 1.5 h at room temperature, followed by 24-h incubation with a polyclonal primary antibody against PV produced in rabbit (1:800, rabbit anti-PV PV27, Swant Swiss Antibodies, RRID:AB_2631173) diluted in blocking solution at 4°C. After rinsing in PBS for 5 × 5 min, slices were incubated with the polyclonal secondary antibody produced in goat (1:200, goat anti-rabbit Alexa Flour 488, ab150077, Abcam) in blocking solution for 5 h at room temperature in darkness. Slices were rinsed 3 × 5 min in PBS and mounted on object slides (Superfrost Plus, Thermo Scientific) and coverslipped with anti-fade mounting medium (Vectashield H-1000, Vector Laboratories).

### Data analysis and statistics

#### ABR

**Figure 1.**
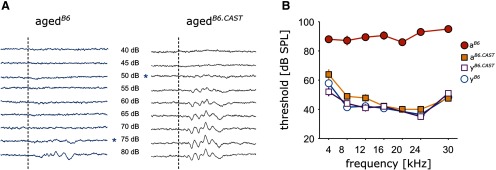
Hearing status of the different groups. ***A***, Examples of averaged ABR traces in response to tone pips at 4 kHz, played back at levels ranging from 40 to 80 dB. The dashed line indicates the onset of a 10-ms tone pip. The asterisks mark the level at which the threshold was set. Left, Individual from the aged^*B6*^ group. Right, Animal from the aged^*B6.CAST*^ group without AHL, both animals were measured at an age of 14 months. ***B***, Mean threshold for all four groups at different sound frequencies. Circles depict B6 animals, squares display animals from the B6.CAST line. Open symbols represent young and filled symbols aged animals (mean ± SEM).

For every stimulus at least 400 artifact free trials were recorded. Thresholds at individual frequencies were determined by eye from the average stimulus-aligned traces at each frequency as the lowest level that evoked an ABR (Fig. 1*A*). If no response could be evoked up to 95 dB, threshold was set to 95 dB, which was the highest level we were able to present without distortions.

#### Histology

**Figure 2.**
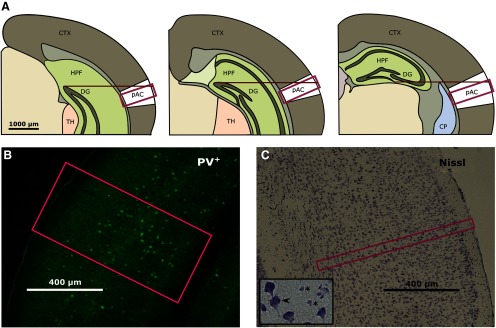
Analysis of histology. ***A***, Identification of the pAC. The pAC was identified by using the hippocampal fissure (HF; DG, dentate gyrus) as a reference structure. Three selected regions along the rostro-caudal axis are shown (from left to right, y coordinate relative to bregma: −3.68, −3.005, −2.48 mm). The landmark was chosen according to the mouse brain reference atlas and centered under the microscope. By following a straight line, the camera section was moved to the cortex (CTX) and the center of pAC was photographed. The magenta rectangle symbolizes the ROI that was used for the counting procedure. For the confirmation of the cortex position, see Extended Data Figure 2-1 and Tsukano et al. (2016). TH: thalamus; CP: caudoputamen. ***B***, Example image with ROI for the counting of PV^+^ neurons. ***C***, Example image with ROI for the counting of Nissl-stained neurons. Detailed image, distinguishing between neurons and other cell types (arrow, neuron; asterisk, non-neuronal cell type).

For quantitative analysis of the PV immunoreactivity in young and old animals (with and without AHL), pAC was photographed at 10 × magnification using an Axioskop 2 MOT Plus (Carl Zeiss Microscopy GmbH) equipped with a camera (Eos 7D, Canon). The pAC was identified by using anatomic landmarks (method adopted from Martin del Campo et al. (2012)) and a mouse brain atlas as reference (Allen Mouse Brain volumetric atlas 2012; https://mouse.brain-map.org). Images were acquired directly above the center by placing the pAC into the middle of the camera section (Fig. 2*A*). Images were exported as TIFF. Neurons and PV^+^ neurons were counted within the region of interest (ROI; 400-µm height for PV slices and 50 µm for Nissl slices; Fig. 2*B*,*C*), which was placed above the center. We previously confirmed the position of auditory cortex in B6.CAST animals (Extended Data Fig. 2-1; Gothner et al., 2019). Counting was performed manually by a person who was blind to the experimental condition using the cell counter plugin in Fiji for non-automated quantification (Schindelin et al., 2012). Only neurons with a clearly identifiable soma were labeled as positive. In Nissl slices, only cells with the neuron-characteristics perikarya and soma staining were counted (Fig. 2*C*). Neurons which were touching the lateral (relative to cortex) border where included, whereas those touching the medial border of the ROI were excluded.

10.1523/ENEURO.0511-19.2020.f2-1 Extended Data Figure 2-1 Confirmation of the position of auditory cortex in B6.cast mice. ***A***, A movable electrode array was implanted, stereotactically positioned at 2.6 mm anterior to bregma. The path of the probe was angled at 24° in order to follow the path indicated by the black line. Arrowheads indicate the positions of the starting point after surgery and the last position that was recorded from in the subject with furthest travel of the probe. Final positions varied from subject to subject. Recordings were obtained from an array of eight tetrodes at each position in awake, unrestrained animals, typically yielding 20–30 units per position. The orange area depicts cortical fields labeled as “auditory areas” according to the Allen mouse brain reference atlas (version 2012), which is based on tracing data. The thick yellow line at the top of the cortex indicates auditory areas in B6/C57 mice as determined by a study using physiological imaging (Tsukano et al., 2016). The red box is our window for counting PV^+^ cells, aligned by anatomical landmarks at 2.6 anterior to bregma. ***B***, Proportion of auditory and non-auditory units recorded at each position along the probe path. Each horizontal sequence of pie charts represents data from a single animal (*n* = 6), the horizontal position of the pie center marks the position of the probe along the path. The grey area depicts the medial-lateral position of the window used for counting PV^+^ cells at 2.6 anterior to bregma. Each pie chart displays the proportion of non-auditory units (white), determined by a stimulus set containing both simple tone and complex naturalistic stimuli. Auditory units were classified as primary (blue) and non-primary (red) based on response latency (<20 ms) to tone stimuli, responsiveness to repeated simple stimuli, and the shape of the frequency tuning curve. Data was collected in the context of another study, for further methodological details see Gothner et al. (2019). Download Figure 2-1, TIF file.

For each animal, at least eight slices (both hemispheres), distributed along the rostro caudal axis were analyzed to calculate the mean number of PV^+^ neurons. The depth of the cortex (from pia to white matter) was labeled in each slice to calculate the mean density of PV^+^ neurons (neurons/mm^2^). The total number of neurons (Nissl) was multiplied by the factor of eight and the mean served as a control for the evaluation of possible cell loss during aging and/or AHL.

All variables (number of neurons, number of PV^+^ neurons and density of PV^+^ neurons) were statistically analyzed (IBM SPSS Statistics version 25, IBM) for the following groups: young (including both, young*^B6^* and young*^B6.CAST^*), aged*^B6^* and aged*^B6.CAST^*. Data were tested for normal distribution and homogeneity of variances before the ANOVA. Effect size was calculated as $=\sqrt{\eta^{2}/1-\eta^{2}}$. Power (1-*β*, see Table 2) was calculated using G*Power (Faul et al., 2007). A one-sided *post hoc* test (Dunnett) was applied according to the hypothesis of a reduction in PV cells in aged animals of both groups (young*^B6/B6.CAST^*> aged*^B6^* and aged*^B6.CAST^*). Because of the small sample size of all groups, the level of significance was set to α = 0.01.

**Table 2:** Statistics

Ref #	Data structure	Parameters tested	Type of test	Power (1-β err prob)	Figure
A	Three independent samples: young*^B6/B6.CAST^* (*n* = 8), aged*^B6^* (*n* = 8), aged*^B6.CAST^* (*n* = 6)	Total number of neurons/ROI	ANOVA	0.3739166	3*A*
B	Three independent samples: young*^B6/B6.CAST^* (*n* = 8), aged*^B6^* (*n* = 8), aged*^B6.CAST^* (*n* = 6)	Number of PV^+^ interneurons/ROI	ANOVA	0.9993094	3*B*
C	Three independent samples: young*^B6/B6.CAST^* (*n* = 8), aged*^B6^* (*n* = 8), aged*^B6.CAST^* (*n* = 6)	Density of PV^+^ interneurons, number of PV^+^/mm^2^	ANOVA	0.8839026	3*C*

Differences in number and density of PV^+^ interneurons might not be observable in the whole sample but could be dependent of the cell position in the dimension of layer or rostro-caudal axis. In order to evaluate the laminar distribution of PV^+^ in the different animal groups, cell coordinates were projected onto the axis between pia and white-matter boarder in each individual slice. Laminar position was calculated as the distance from the pia relative to pia-white matter distance. Laminar position was binned in 10 equally sized windows and averaged to obtain a laminar distribution for each animal. Subsequently, mean distributions for each group were calculated.

In order to obtain a position along the rostro-caudal axis, slices were aligned to the Allen Mouse Brain volumetric atlas (Allen Mouse Brain volumetric atlas 2012; https://mouse.brain-map.org) and referenced to bregma coordinates. Slice positions were binned in 204 µm windows ranging from 2458 to 3681 µm caudal relative to bregma. All cells within a specific laminar bin and on slices within each rostro-caudal bin were counted to obtain distributions of PV^+^ neurons along the rostral-caudal and laminar axes for each animal group.

## Results

### Aged animals from the B6.CAST line do not show AHL

We aimed to test whether the previously reported decline in PV immunoreactivity in the auditory cortex of C57/B6 mice could be a result of their early onset AHL. To this end we compared aged wild-type C57/B6 mice (aged*^B6^*) with a control group of the mutant C57B6.CAST-Cdh23^Ahl+^ (aged*^B6.CAST^*) that is not susceptible to AHL.

In a first step, we therefore confirmed the difference in AHL between the two aged groups (Fig. 1). In aged*^B6^* we found an increase of the threshold over the entire tested frequency range (Fig. 1*B*) with the mean threshold increasing from 44.6 ± 7.2-dB SPL (mean ± SD, *n* = 6) to 89.8 ± 3.2-dB SPL (*n* = 7). For the B6.CAST group, we could observe almost no change in thresholds across all frequencies (mean thresholds young 44.0 ± 7.0-dB SPL, *n* = 6 vs 47.2 ± 8.3-dB SPL in the aged*^B6.CAST^* group, *n* = 9). ABR thresholds for young animals of the two lines were not different (44.6 ± 7.2 vs 44.0 ± 7.0-dB SPL). Consequently, data from young*^B6^* and young*^B6.CAST^* have been pooled in the immunohistochemical experiments.

### Aging causes a reduction in PV immunoreactivity in the pAC of mice with and without AHL

In the present study, we investigated the number and density of PV^+^ neurons in the pAC of young*^B6/B6.CAST^* (both lines pooled) and old mice with and without the presence of AHL, previously determined by the ABR measurements.

**Figure 3.**
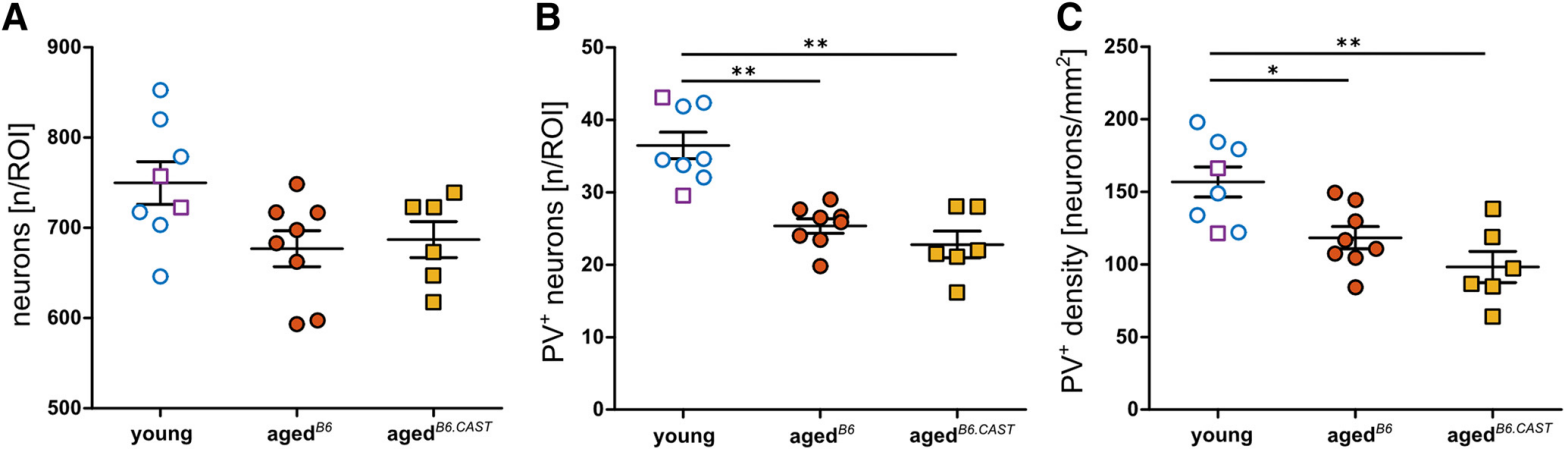
Total number of neurons and PV^+^ immunoreactivity of the different groups. Circles depict B6 animals, squares display animals from the B6.CAST line. Open symbols represent young and filled symbols aged animals (mean ± SEM), significance values are displayed by number of asterisks: *p < 0.01; **p < 0.001. ***A***, Mean total number of neurons/ROI. No significant difference could be revealed for the total number of neurons. ***B***, Mean number of PV^+^ neurons/ROI. When compared with young*^B6/B6.CAST^*, both aged*^B6^* and aged*^B6.CAST^* show a significant decrease in number of positive neurons. ***C***, Mean PV^+^ density. Young animals show a significant higher density of PV^+^ neurons compared to aged animals of both groups.

Possibly, a decline in total number of neurons may be stronger in one of the aged groups which could confound the interpretation of the total number of PV^+^ cells. To ensure that our results of the immunohistochemical verification were not influenced by a global decline in cortical neurons, the total number of neurons was investigated using a Nissl protocol. A mild, non-significant decrease of neurons was present in aged*^B6.CAST^* and aged*^B6^* compared with young*^B6/B6.CAST^* (*F*_(2,19)_ = 3.523; *p* = 0.05, ANOVA; *f *=* *0.61, 1-*β* = 0.374; *post hoc* pair-wise comparison Dunnett, one-sided, young*^B6/B6.CAST^* > aged*^B6^*: *p* = 0.021; young*^B6/B6.CAST^* > aged*^B6.CAST^*: *p* = 0.056; Fig. 3*A*).

The immunohistochemical verification has been performed to investigate whether (1) a decline of PV immunoreactivity exists during aging and (2) whether it is stronger in animals suffering from progressive AHL. A significant decrease in number of PV^+^ neurons could be observed for aged*^B6.CAST^* and aged*^B6^* groups (*F*_(2,19)_ = 1.182; *p* < 0.001, ANOVA; *f *=* *1.49, 1-*β* = 0.9993; Fig. 3*B*). The one-sided pairwise comparison to young*^B6/B6.CAST^* animals revealed a strong difference for both, young*^B6/B6.CAST^* versus aged*^B6^* and young*^B6/B6.CAST^* versus aged*^B6.CAST^* (*p* < 0.001).

Additionally, we investigated the density of PV^+^ neurons by taking the cortical depth of each analyzed imaged into account (400-µm ROI width × cortical depth, calculated as neurons/mm^2^; Fig. 3*C*). The density of PV^+^ neurons/mm^2^ differed significantly (ANOVA, *F*_(2,19)_ = 9.301; *p* < 0.01; *f *=* *0.99, 1-*β* = 0.884). Similar to the absolute counts, we found a significant lower density in both aged*^B6^* versus young*^B6/B6.CAST^* (*p* < 0.01) and aged*^B6.CAST^* versus young*^B6/B6.CAST^* (*p* = 0.001).

**Figure 4.**
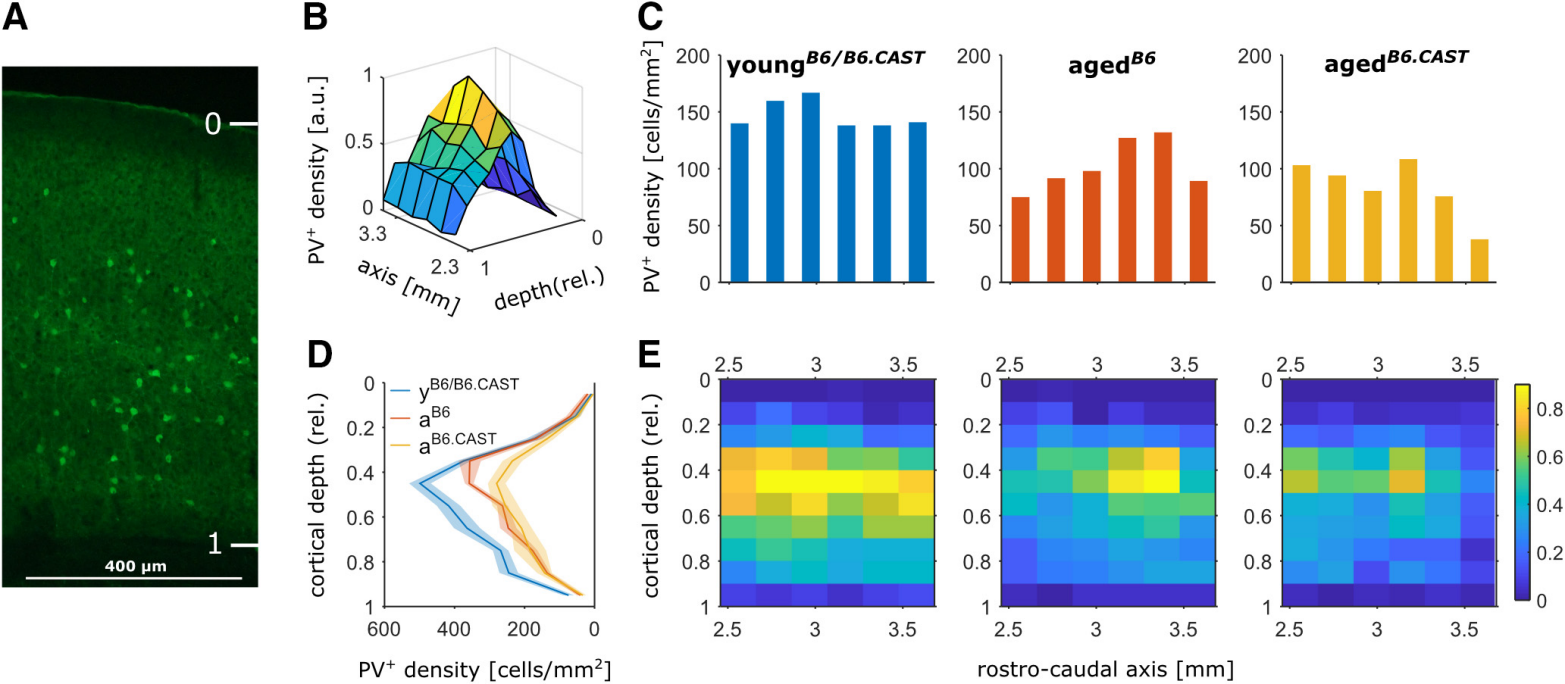
Distribution of PV^+^ neurons across layers and along the rostro-caudal axis. ***A***, Illustration of the relative laminar mapping of the cell positions within the single slices. The pia was set to depth = 0 and the border to white matter was set to depth = 1. ***B***, Population overview. Relative density of PV^+^ neurons resolved by the rostro-caudal position of slices and cortical depth of cell body is shown. Here, density was averaged over all slices from all animals to reveal an age-independent overview about the general PV pattern in the pAC. Density was normalized to the maximum of the distribution. ***C***, Cell density resolved by rostro-caudal position of the slices (relative to bregma). Each bar depicts mean density for all slices within the respective bin along the rostro-caudal axis. Left, Young animals of both lines. Middle, aged*^B6^* group. Right, aged*^B6.CAST^* group. ***D***, Laminar distribution of PV^+^ neurons in the three groups (mean ± SEM). Positions are relative to the pia (see ***A***). ***E***, Relative density of PV^+^ neurons in the slices resolved by rostro-caudal position and cortical depth in the three groups. Color-coded densities are normalized to the maximum mean density in the young group (left, young*^B6/B6.CAST^*; middle, aged*^B6^*; right, aged*^B6.CAST^*). Plots (***B–E***) were generated using MATLAB (version R2018b; The MathWorks Inc.).

Differences in number and density of PV^+^ interneurons might not be observable in the whole sample but could be dependent of the cell position in two dimensions: layer positions defined as cortical depth and along the rostro-caudal axis. In order to receive a detailed description of the immunoreactivity pattern, we performed an analysis of PV immunoreactivity in in these two dimensions in the pAC of all animals. We observed a clear layer dependence of PV^+^ neurons revealed by the analysis of the density as a factor of cortical depth, with a strong peak in deep (subgranular) layers (Fig. 4*A*). The largest density was present in the middle layers of pAC (maximum just below Layer IV). This layer dependence occurred with a nearly uniform distribution across the rostra-caudal axis of pAC (data of all animals, Fig. 4*B*). Subsequently, we analyzed layer dependence and rostro-caudal distribution for the factor of age and AHL. We could not observe a robust overall pattern of reduction along the rostro-caudal axis, but slightly higher reduction of PV^+^ neurons in the rostral portion of pAC in aged*^B6^* compared with young and aged*^B6.CAST^* (Fig. 4*C*). In contrast, the reduction was stronger in the caudal region in aged animals without AHL (aged*^B6.CAST^*). When comparing groups according to the factor of cortical depth (Fig. 4*D*), the reduction across lamina was uniform in both, aged*^B6.CAST^* and aged*^B6^*. PV density in the middle layers was mildly more reduced for animals from the aged*^B6.CAST^*, group than from aged*^B6^*. In summary, the combined pattern of reduction (Fig. 4*E*) across laminar and rostro-caudal axis appears to be very similar for aged animals with AHL (aged*^B6^*) and without (aged*^B6.CAST^*).

## Discussion

We aimed to reveal whether a decrease in PV immunoreactivity during aging is stronger in the presence of AHL, as previously indicated (de Villers-Sidani et al., 2010; Martin del Campo et al., 2012; Brewton et al., 2016). On that account, we investigated the hearing thresholds and the PV immunoreactivity in the pAC of aged animals from a mouse line with progressive, early onset AHL (C57BL/6J) and from a congenic strain which is not vulnerable to AHL (C57B6.CAST-Cdh23*^Ahl+^*), in comparison with young animals. The main outcome of this study is a decline in PV immunoreactivity in the pAC of aged mice, regardless of AHL. Although AHL could be confirmed by our ABR measurements for the aged*^B6^* animals (Fig. 1), both aged strains showed a similar reduction of PV^+^ positive interneurons in pAC in both number and density (Fig. 3). The pattern of reduction across the rostro-caudal axis and across cortical layers was similar in both strains (Fig. 4).

### Specificity of reduction of PV cells

We investigated PV immunoreactivity in the pAC of all experimental groups by an indirect, immunofluorescent approach, using a primary antibody against PV. Additionally, as a control, the total number of neurons was evaluated to test for possible unequal cell loss between aged groups, following a standard Nissl protocol. Reduced PV immunoreactivity and thus a smaller number of positive cells may have been the results of a global loss of neurons for the aged groups compared with young animals and may be more severe in animals suffering from AHL. In our study, we found the total number of neurons in the pAC to be decreased in both aged groups, albeit not significantly (Fig. 3*A*). This mild, non-significant decrease in number of neurons for aged animals has been previously reported, at least for the rat auditory cortex (Burianova et al., 2015). In relation to our data from the evaluation of PV immunoreactivity (Fig. 3*B*,*C*), it can be concluded that a decreased number of PV^+^ interneurons is not solely based on a cortical neuronal decline in only one of the experimental groups. However, the identity of the lost neurons (reflected by the mild decrease in number of neurons) cannot be clearly defined with our experimental approach. It remains to be shown whether PV^+^ neurons remain intact but display a reduced protein expression or if the density of these specific inhibitory interneurons is decreased.

### Reduction of PV^±^ interneurons is present in aged animals with preserved hearing thresholds

Regarding the number and density of PV^+^ neurons, aged*^B6.CAST^* and aged*^B6^* show a decrease of both. Indeed, a reduction in PV immunoreactivity seems to be dominated by the factor age rather than by hearing loss. This is in line with results from other studies, reporting decreased inhibitory properties in the aged auditory cortex independent of hearing status (Ueno et al., 2018; Kessler et al., 2020) and a reduction in PV^+^ neurons in other cortical areas, like somatosensory or motor (Miettinen et al., 1993).

One main reason for controversial results might be strong strain differences of PV immunoreactivity levels in animals. These differences are not only present on the molecular but also on the physiological level (Brewton et al., 2016; Bowen et al., 2019). All work that has been done so far to reveal the influence of AHL on the PV immunoreactivity pattern used either two different strains of laboratory rodents with/without the development of AHL (Ouda et al., 2008; Brewton et al., 2016) or compared old versus young animals of the same strain (de Villers-Sidani et al., 2010; Martin del Campo et al., 2012). It is possible that not only physiological properties differ between strains, but also the development of PV immunoreactivity patterns over time. In our study, the only difference between the two mouse strains used is the wild-type allele of *Cdh23* in aged*^B6.CAST^* animals, eliminating the possibility of general strain differences in basal PV immunoreactivity patterns. However, it cannot be ruled out that patterns of PV immunoreactivity in the central auditory system depend on the genetic, peripherally acting cause of AHL. As the cause in our hearing impaired aged^B6^ animals, the missense mutation of *Cdh23* is believed to result in a defective encoded protein that acts as a component of the stereocilia tip links in hair cells. The defect can cause a weakening of the tip links over time, which may result in progressively impaired mechanotransduction in the aging individual (Kazmierczak et al., 2007; Schwander et al., 2009; Johnson et al., 2010). Whether our results can be directly transferred to other mutations needs to be further investigated, e.g., in mutations of *Sod1*, which encodes for a defective superoxide dismutase, resulting in increased oxidative stress and therewith hair cell loss in the inner ear (McFadden et al., 2001; Jiang et al., 2007; Johnson et al., 2010). However, our results can be interpreted as a first step toward a better understanding about the central molecular alterations in Cdh23-mediated presbycusis.

### Pattern of PV immunoreactivity along the rostro-caudal axis and cortical depth

To get an overall impression of the neuronal PV pattern in the pAC of young, aged, and aged mice expressing hearing loss, we provide a two-dimensional descriptive analysis of the rostro-caudal and layer-dependent distribution of PV^+^ interneurons. Our data indicate an overall similar rostro-caudal distribution for all tested groups. However, the loss of PV immunoreactivity varies considerably between adjacent individual rostro-caudal sections (Fig. 4*C*) and may show differences between the groups. For a more fine-grained, unbiased evaluation of differences between immunoreactivity patterns, it should be considered to analyze multiple samples per animal along the rostro-caudal axis of the structure of interest as done in this study. Small sample sizes confined to narrow rostro-caudal sections could lead to false-positive differences that might disappear when analyzing a sample size distributed along the rostro-caudal axis or vice versa.

The PV density as a function of cortical depth peaks in middle layers, with a small population of neurons in the deeper and the fewest number in the superficial layers (Fig. 4*D*) as previously revealed by others (del Río et al., 1994; Martin del Campo et al., 2012; Brewton et al., 2016; Ueno et al., 2018). Regarding age and hearing status, we could not detect a difference between distributions of an aged*^B6.CAST^* or an aged*^B6^* animal. Thus, an altered density of PV^+^ neurons along the cortical depth is related to the factor age and remains unaltered by hearing status, similar to what we observed for PV immunoreactivity along the rostro-caudal axis.

### Reduced PV immunoreactivity in the pAC, a sign of central molecular aging in the mouse model

The C57BL/6 mouse is commonly used as an animal model to study presbycusis due to its progressive, early onset AHL which is the result of the degenerating basal portion of the cochlear (Ison et al., 2007; Park et al., 2010; Martin del Campo et al., 2012). This process can be confirmed by analyzing ABRs, and it has been shown to become histologically detectable by the age of three months (Park et al., 2010). In parallel, a second aging-induced process seem to act on the central (auditory) system in form of reduced PV immunoreactivity in the inhibitory network of the cortex. Our data indicates that this process is independent of declined hearing sensitivity and should rather be interpreted as a sign of central molecular aging. The consequences of reduced intraneuronal protein levels in inhibitory system are not fully understood yet. Hence, it remains controversial to what extent a reduction of PV immunoreactivity affects inhibition and therewith central sensory processing in the (auditory) cortex.

PV itself as a calcium-binding protein is believed to serve the distinct function of buffering intracellular Ca^2+^, enabling the neuron to fire rapid spike trains and protecting it from toxic intracellular calcium levels (Ferguson and Gao, 2018; Fairless et al., 2019). This might support the unique function of this neuronal subtype: PV^+^ interneurons are known for their remarkable fast spiking phenotype with a local widespread of activity regulating contacts to nearly every pyramidal neuron in their surrounding (Kawaguchi and Kubota, 1997; Packer and Yuste, 2011; Ferguson and Gao, 2018). On a functional level, this organization principle allows for strong feedback and feedforward inhibition with a precision in the millisecond range (Cardin, 2018; Hu et al., 2014). A change in the physiology of PV^+^ cells may be particularly impactful in the auditory cortex, where precise modulation of sensory input is of great importance as it sharpens spike timing, shapes receptive fields, provides gain control and is involved in the generation of network oscillations (Wehr and Zador, 2003; Sohal et al., 2009; Moore and Wehr, 2013; Hu et al., 2014; Gothner et al., 2019). In that context, a reduced PV immunoreactivity could result in declined inhibitory properties in cortical circuits, which seems to be independent of AHL.

An additional potential role of PV that is discussed in current research is its involvement in (synaptic) plasticity: PV seems to prevent cumulative facilitation and maintains the strength of the synapse near its resting level (Caillard et al., 2000; Tripodi et al., 2018). Additionally, a few studies indicate that the PV immunoreactivity pattern cannot be interpreted as a static rather than an ongoing plastic process, influenced by environmental factors as previously demonstrated by de Villers-Sidani et al. (2010), who showed that decreased PV immunoreactivity in the aged auditory cortex of rats can be recovered by auditory training.

Given the importance of PV^+^ inhibition and the strong reduction of PV immunoreactivity in the aging auditory cortex as shown here, further research is urgently needed to reveal the actual consequences of age-related reduction of PV immunoreactivity on inhibitory circuits on a physiological level. However, given the results presented here, physiological and possibly perceptual consequences of PV^+^ reduction in pAC will have to be seen as a result of general, molecular aging in the auditory cortex instead of being restricted to individuals suffering from AHL.

## Synthesis

Reviewing Editor: Helene Benveniste, Yale University School of Medicine Decisions are customarily a result of the Reviewing Editor and the peer reviewers coming together and discussing their recommendations until a consensus is reached. When revisions are invited, a fact-based synthesis statement explaining their decision and outlining what is needed to prepare a revision will be listed below. The following reviewer(s) agreed to reveal their identity: Chandresh Gajera, Karl Herrup.

Synthesis of review:

The reviewers express mixed opinions on whether or not the results represent a significant advance to the field of understanding and dissect effect of aging and aging-releted hearing loss on the PV immunoreactivity in  the primary auditory cortex of mice. However, both reviewers find the results intriguing.

One reviewer expresses minor concerns but regards the results more as ‘proof of concept to dissect ageing and not aging related hearing loss and caution that the results may not hold up in other mutations that leads to hearing loss or under different genetic background. The other reviewer has significant technical concerns regarding rigor of the methods including 1) cell counting; 2) signal-to-noise of parvalbumin staining; 3) ability to distinguish neurons from other cell types and 4) morphometric delineation of the auditory cortex. These major concerns will need to be addressed in the revision.

Comments from Reviewer 1:

Manuscript entitled "Aging but not age-related hearing loss dominates the decrease of parvalbumin immunoreactivity in the primary auditory cortex of mice" by undisclosed authors are well and logically written with detailed discussion.

Authors attempted to dissect effect of aging and AHL on the PV immunoreactivity in the primary auditory cortex (PAC) of mice, by using a mouse line (C57BL/6) and the mutant C57B6.CAST-Cdh23Ahl+, there after compared young and old for their hearing status by using auditory brainstem response measurements and an immunohistochemical evaluation of the PV immunoreactivity patterns. Overall authors have done careful genetic experiment in young and aging mice to demonstrate their claim. However authors shall discuss in discussion that results that are results valid here in C57B6.CAST-Cdh23Ahl+ as proof of concept to dissect ageing and not aging related hearing loss for decrease in PV immunoreactivity.... however one may be need to be cautious that extension of results may or may not hold valid in other mutations that leads to hearing loss or under different genetic background. It also remains to be seen that whether the PV cells are lost/decreased or cells are their but it lost/decreased the expression of PV, and hence loss in immunoreactivity.

Minor observations:

In Document "111294_0_merged_1575824251"

Line 5 " ore" and Line 39 "largetst" needs edit

Line 48: word "disentangle" may be changed to "dissect" or " resolve"

Line 56 and 57 : "XX" and "XXXX" need to be replaced with appropriate words

Figure 4B: How these figures were generated? Please include in method section and also include the underlying raw data used to generate this figure in table format. (at appropriate place or in supplement)

Reviewer 2 comments:

The manuscript addresses an interesting question of auditory function and aging. The authors test the question of whether the loss of parvalbumin inhibitory neurons that is seen in the C57BL/6 strain of mouse - a strain that suffers from age-related hearing loss - is caused by the hearing loss itself or is just a natural part of the aging process. Using quantitative methods to analyze both parvalbumin immunocytochemistry and Nissl staining for total neurons, they conclude that in fact the loss of the parvalbumin neurons is independent of hearing loss.

The work suffers from a number of problems with the morphometric analysis that lower confidence in the strength of the conclusions. The definition of the primary auditory cortex is one issue. The landmarks used were worked out for a few strains, but the CAST line is more recently derived from wild stock and is genetically distinct. Without careful validation, it is not certain that the same landmarks can be uniformly applied. This could be solved by anterograde labeling to ensure the fidelity of the boundaries assigned. Alternatively, a larger region of cortex could be sampled.

Cell counts using Fiji/ImageJ need to be carefully validated. The software is useful and powerful, but compromises need to be made in any application (especially when signal to noise is low - see below). Manual counts should be done on a select set of samples to validate the automated results.

The size of the object counted is not adequately controlled for. More large cells than small cells will appear to be present in a 25mcm section even if their density is the same. The classic way to correct for this is to apply Abercrombie's correction: N = n(t/(t+d)). Where N is the true number of cells, n is the number of cells counted, t is the section thickness, and d is the diameter of the object counted. There are more sophisticated methods involving stereology, but some effort must be made to account for this.

The identity of the cells is not clear. The immunostaining in Figures 1 and 4 suggests that the signal to noise of the parvalbumin staining is not high. More details are needed to assure the reader that the counts are truly representative. The Nissl counts are equally problematic. The Nissl stain will label all of the cell types in the brain. Did the authors make any attempt to distinguish neurons from astrocytes and other non-neuronal cells? Finally, the Y-axes in Figure 3A and B are unclear. The implication is that "n" is the absolute number of neurons, but this is not plausibly true. There are certainly more than 750 neurons (cells) in the entire auditory cortex for example. This issue must be clarified for the reader to be able to judge the results

Given these issues, the work is difficult to fully evaluate. The impression is strong that the authors are correct in their conclusions, but the level of rigor in the analysis does not allow for this impression to begin to approach certainty.
