# Supplementary figures and images for: Aging But Not Age-Related Hearing Loss Dominates the Decrease of Parvalbumin Immunoreactivity in the Primary Auditory Cortex of Mice
Source: eNeuro. 2020 May 8;7(3):ENEURO.0511-19.2020. doi: 10.1523/ENEURO.0511-19.2020 (PMC7210488; doi:10.1523/ENEURO.0511-19.2020)

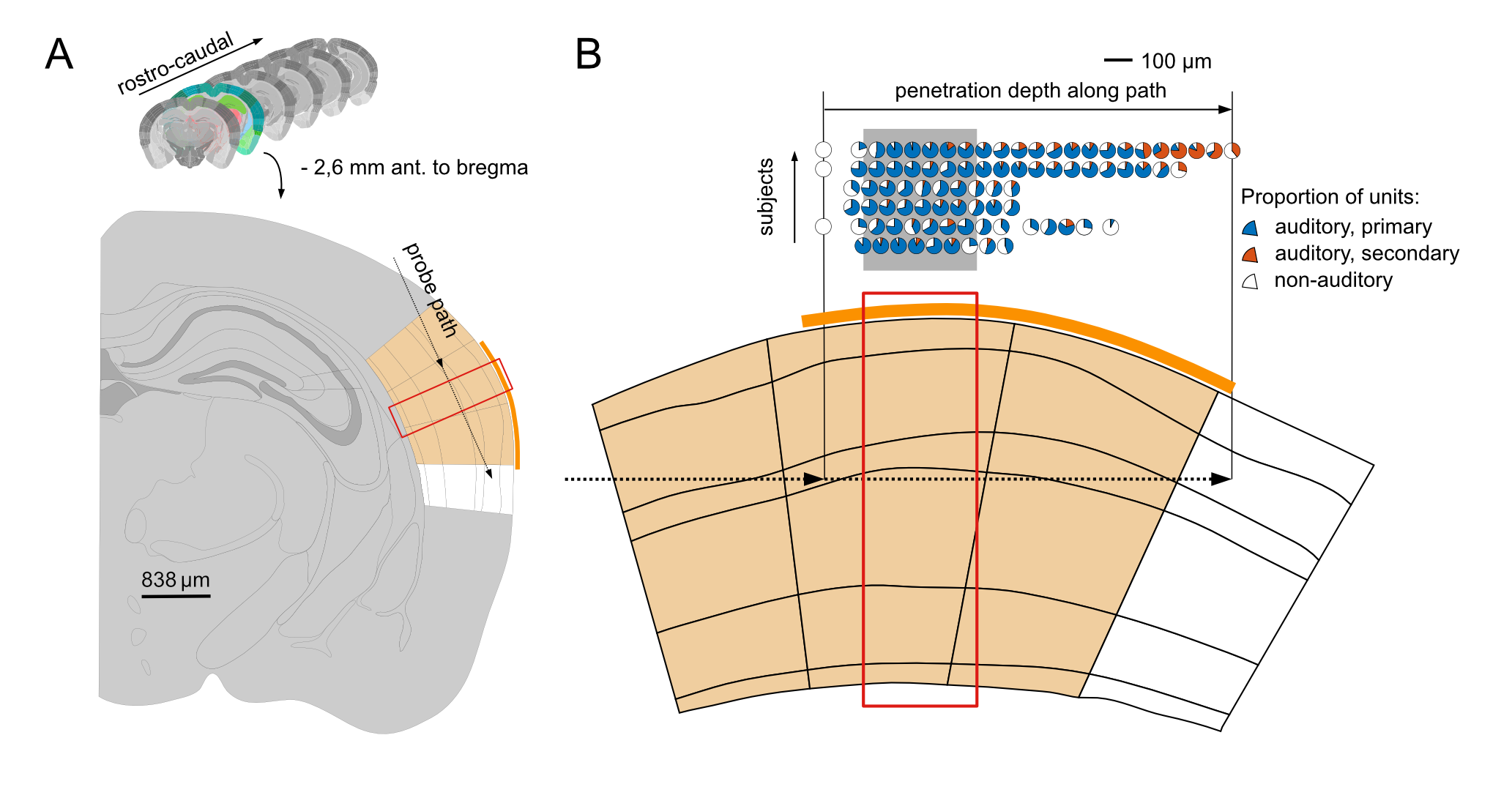

Supplement: Extended Data Figure 2-1 — Confirmation of the position of auditory cortex in B6.cast mice. A, A movable electrode array was implanted, stereotactically positioned at 2.6 mm anterior to bregma. The path of the probe was angled at 24° in order to follow the path indicated by the black line. Arrowheads indicate the positions of the starting point after surgery and the last position that was recorded from in the subject with furthest travel of the probe. Final positions varied from subject to subject. Recordings were obtained from an array of eight tetrodes at each position in awake, unrestrained animals, typically yielding 20–30 units per position. The orange area depicts cortical fields labeled as “auditory areas” according to the Allen mouse brain reference atlas (version 2012), which is based on tracing data. The thick yellow line at the top of the cortex indicates auditory areas in B6/C57 mice as determined by a study using physiological imaging (Tsukano et al., 2016). The red box is our window for counting PV+ cells, aligned by anatomical landmarks at 2.6 anterior to bregma. B, Proportion of auditory and non-auditory units recorded at each position along the probe path. Each horizontal sequence of pie charts represents data from a single animal (n = 6), the horizontal position of the pie center marks the position of the probe along the path. The grey area depicts the medial-lateral position of the window used for counting PV+ cells at 2.6 anterior to bregma. Each pie chart displays the proportion of non-auditory units (white), determined by a stimulus set containing both simple tone and complex naturalistic stimuli. Auditory units were classified as primary (blue) and non-primary (red) based on response latency (<20 ms) to tone stimuli, responsiveness to repeated simple stimuli, and the shape of the frequency tuning curve. Data was collected in the context of another study, for further methodological details see Gothner et al. (2019). Download Figure 2-1, TIF file. [file enu-eN-NWR-0511-19-s02.tif]
